# The *NOD2* Single Nucleotide Polymorphisms rs2066843 and rs2076756 Are Novel and Common Crohn's Disease Susceptibility Gene Variants

**DOI:** 10.1371/journal.pone.0014466

**Published:** 2010-12-30

**Authors:** Jürgen Glas, Julia Seiderer, Cornelia Tillack, Simone Pfennig, Florian Beigel, Matthias Jürgens, Torsten Olszak, Rüdiger P. Laubender, Maria Weidinger, Bertram Müller-Myhsok, Burkhard Göke, Thomas Ochsenkühn, Peter Lohse, Julia Diegelmann, Darina Czamara, Stephan Brand

**Affiliations:** 1 Department of Medicine II - Grosshadern, Ludwig-Maximilians-University, Munich, Germany; 2 Department of Preventive Dentistry and Periodontology, Ludwig-Maximilians-University, Munich, Germany; 3 Department of Human Genetics, RWTH (Rheinisch-Westfälische Technische Hochschule), Aachen, Germany; 4 Division of Gastroenterology, University of Leuven, Leuven, Belgium; 5 Division of Gastroenterology, Hepatology and Endoscopy, Brigham and Women's Hospital, Harvard Medical School, Boston, Massachusetts, United States of America; 6 Institute of Medical Informatics, Biometry and Epidemiology (IBE), Ludwig-Maximilians-University, Munich, Germany; 7 Max-Planck-Institute of Psychiatry, Munich, Germany; 8 Institute of Clinical Chemistry - Grosshadern, Ludwig-Maximilians-University, Munich, Germany; Erasmus University Medical Center, Netherlands

## Abstract

**Background:**

The aims were to analyze two novel *NOD2* variants (rs2066843 and rs2076756) in a large cohort of patients with inflammatory bowel disease and to elucidate phenotypic consequences.

**Methodology/Principal Findings:**

Genomic DNA from 2700 Caucasians including 812 patients with Crohn's disease (CD), 442 patients with ulcerative colitis (UC), and 1446 healthy controls was analyzed for the *NOD2* SNPs rs2066843 and rs2076756 and the three main CD-associated *NOD2* variants p.Arg702Trp (rs2066844), p.Gly908Arg (rs2066847), and p.Leu1007fsX1008 (rs2066847). Haplotype and genotype-phenotype analyses were performed. The SNPs rs2066843 (p = 3.01×10^−5^, OR 1.48, [95% CI 1.23-1.78]) and rs2076756 (p = 4.01×10^−6^; OR 1.54, [95% CI 1.28-1.86]) were significantly associated with CD but not with UC susceptibility. Haplotype analysis revealed a number of significant associations with CD susceptibility with omnibus p values <10^−10^. The SNPs rs2066843 and rs2076756 were in linkage disequilibrium with each other and with the three main CD-associated *NOD2* mutations (D'>0.9). However, in CD, SNPs rs2066843 and rs2076756 were more frequently observed than the other three common *NOD2* mutations (minor allele frequencies for rs2066843 and rs2076756: 0.390 and 0.380, respectively). In CD patients homozygous for these novel *NOD2* variants, genotype-phenotype analysis revealed higher rates of a penetrating phenotype (rs2076756: p = 0.015) and fistulas (rs2076756: p = 0.015) and significant associations with CD-related surgery (rs2076756: p = 0.003; rs2066843: p = 0.015). However, in multivariate analysis only disease localization (p<2×10^−16^) and behaviour (p = 0.02) were significantly associated with the need for surgery.

**Conclusion/Significance:**

The *NOD2* variants rs2066843 and rs2076756 are novel and common CD susceptibility gene variants.

## Introduction

Crohn's disease (CD) and ulcerative colitis (UC) are chronic inflammatory bowel diseases (IBD) characterized by an exaggerated immune response of the intestinal mucosa and a dysfunctional epithelial barrier [1], [2], [3], [4]. The identification of nucleotide-binding oligomerization domain 2 (*NOD2,* GeneID: 64127*)*, also known as caspase recruitment domain-containing protein 15 (*CARD15*) as the first susceptibility gene in CD in 2001 [5], [6] has provided significant new insights in the pathogenesis of IBD focusing on the genetic background of innate immune response and interaction with bacterial antigens [7], [8], [9], [10]. Most recently, genome-wide association studies and subsequent replication studies have provided further insights into IBD pathogenesis by identification and confirmation of susceptibility genes such as the interleukin-23 receptor (*IL23R*) [11], [12], *SLC22A4/5* [13] and *ATG16L1* [14] (autophagy-related 16-like 1) gene.

NOD2 represents a cytoplasmatic protein and functions mainly as a NF-κB pathway activating sensor for bacterial muramyl dipeptide (MDP) found in the cell wall of Gram-positive and Gram-negative bacteria [8], [15], [16], [17]. In addition, NOD2 seems to be a negative regulator of Toll-like receptor 2-mediated T helper cell type 1 responses and modulates ileal expression of antimicrobial peptides such as alpha-defensins and the expression of proinflammatory cytokines and chemokines in the intestinal mucosa [18], [19], [20]. The identification of *NOD2* as a susceptibility gene for CD therefore suggests an important role of genetically determined enteric bacteria-host interactions and an inappropriate activation of the mucosal immune system in IBD. Large genotype-phenotype analyses by us [21], [22] and others [23], [24], [25] also demonstrated a significant association of *NOD2* variants with ileal involvement, stricturing phenotype and early disease onset in CD patients.

The *NOD2* gene is located on chromosome 16q in the IBD1 locus and contains 11 constant exons and a twelfth alternatively spliced exon in the 5′-region. So far, three main *NOD2* variants, which include two amino acid substitutions, p.Arg702Trp encoded by exon 4, and p.Gly908Arg encoded by exon 8, and the frameshift mutation p.Leu1007fsX1008 located in exon 11, were identified to be overrepresented in CD patients. There is also evidence for further *NOD2* variants being involved in IBD pathogenesis as demonstrated by us [26] and others in recent association studies [18], [19], [20]. However, the extent of disease modification or phenotypic consequences of other *NOD2* variants such as the SNPs rs2066843 and rs2076756 have not been investigated so far.

We therefore genotyped 2700 individuals of Caucasian origin and performed a large and detailed genotype-phenotype analysis for the *NOD2* variants rs2066843 and rs2076756 analyzing the influence of these variants on the disease susceptibility and phenotype of patients with CD and UC.

## Materials and Methods

### Study population

The study population (n = 2700) was comprised of 1254 IBD patients of Caucasian origin including 812 patients with CD, 442 patients with UC, and 1446 healthy, unrelated controls. The patients were recruited in two cohorts; the discovery sample was recruited from the University Hospital Munich-Grosshadern and comprised 519 CD patients, 232 UC patients and 770 controls, while the replication cohort recruited from the University Hospitals Bochum and Munich-Innenstadt consisted of 293 CD patients, 210 UC patients and 676 controls**.** Patients with indeterminate colitis were excluded from the study. All individuals gave written, informed consent prior to the study. The study was approved by the local Ethics committee and adhered to the ethical principles for medical research involving human subjects of the Helsinki Declaration. Phenotypic parameters were collected blind to the results of the genotype analysis and included demographic and clinical data (behaviour and anatomic location of IBD, disease-related complications, surgical or immunosuppressive therapy). Two senior gastroenterologists analyzed data which were recorded by patient charts analysis and a detailed questionnaire based on an interview at time of enrolment. For the analysis of demographic and phenotypic data, the diagnosis of CD and UC was related to established international guidelines based on endoscopic, radiological, and histopathological parameters [27]. CD patients were classified according to the Montreal classification [28] including age at diagnosis (A), location (L), and behaviour (B) of disease. In patients with UC, anatomic location was also assessed in accordance to the Montreal classification based on the criteria ulcerative proctitis (E1), left-sided UC (distal UC; E2), and extensive UC (pancolitis; E3). The clinical characteristics of the IBD study population are summarized in Table 1.

**Table 1:** Demographic characteristics of the IBD study population.

	Crohn's diseasen = 812	Ulcerative colitisn = 442	Controlsn = 1446
**Gender**Male (%)Female (%)	49.051.0	48.151.9	63.136.9
**Age** (yrs)Mean ± SDRange	39.4±13.210-80	41.7±14.67–85	46.1±10.618–71
**Body mass index**Mean ± SDRange	23.1±4.113–40	23.9±4.215–41	
**Age at diagnosis** (yrs)Mean ± SDRange	27.5±11.57–71	32.1±13.59–81	
**Disease duration** (yrs)Mean ± SDRange	12.2±8.50–44	11.0±7.81–40	
**Positive family history of IBD** (%)	16.2	16.1	

### DNA extraction and NOD2 genotyping

Genomic DNA was isolated from peripheral blood leukocytes by standard procedures using the DNA blood mini kit from Qiagen (Hilden, Germany). Genotyping of the *NOD2* variants p.Arg702Trp (rs2066844), p.Gly908Arg (rs2066847), and p.Leu1007fsX1008 (rs2066847) were performed as described previously [26] (primer and probe sequences are available on request). The *NOD2* SNPs rs2066843 and rs2076756 were genotyped by PCR and melting curve analysis using a pair of fluorescence resonance energy transfer (FRET) probes, a sensor and an anchor probe, respectively, in a LightCycler 480 Instrument (Roche Diagnostics, Mannheim, Germany). The donor fluorescent molecule (fluorescein) at 3′-end of the sensor probe in case of rs2066843 or the anchor probe in case of rs2076756, respectively, is excited at its specific fluorescence excitation wavelength (533 nm) and the energy is transferred to the acceptor fluorescent molecule at the 5′-end of the anchor probe in case of rs2066843 (LightCycler Red 640) or the sensor probe in case of rs2076756 (LightCycler Red 670). The specific fluorescence signal emitted by the acceptor molecule is detected by the optical unit of the LightCycler 480 Instrument. The sensor probe is exactly matching to one allele of each SNP, whereas in the case of the other allele there is a mismatch resulting in a lower melting temperature. The total volume of the PCR was 5 µl containing 25 ng of genomic DNA, 1 x Light Cycler 480 Genotyping Master (Roche Diagnostics), 2.5 pmol of each primer and 0.75 pmol of each FRET probe (TIB MOLBIOL, Berlin, Germany). The PCR comprised an initial denaturation step (95°C for 10 min) and 45 cycles (95°C for 10 sec, primer annealing temperature as given in Supplemental Table S1 for 10 sec, 72°C for 15 sec). The melting curve analysis comprised an initial denaturation step (95°C for 1 min), a step rapidly lowering the temperature to 40°C and holding for 60 sec, and a heating step slowly (1 acquisition/°C) increasing the temperature up to 95°C and continuously measuring the fluorescence intensity. The results of melting curve analysis had been confirmed by analyzing samples representing all possible genotypes using sequence analysis. For sequencing, the total volume of the PCR was 100 µl containing 250 ng of genomic DNA, 1 x PCR-buffer (Qiagen, Hilden, Germany), a final MgCl_2_ concentration of 1.5 mM, 0.5 mM of a dNTP-Mix (Sigma, Steinheim, Germany), 2.5 units of HotStar Plus Taq™ DNA polymerase (Qiagen) and 10 pmol of each primer (TIB MOLBIOL, Berlin, Germany). The PCR used for sequencing comprised an initial denaturation step (95°C for 5 min), 35 cycles (denaturation at 94°C for 30 sec, primer annealing at 60°C for 30 sec, extension at 72°C for 30 sec) and a final extension step (72°C for 10 min). The PCR products were purified using the QIAquick PCR Purification Kit (Qiagen) and sequenced by a commercial sequencing company (Sequiserve, Vaterstetten, Germany). All sequences of primers and FRET probes and primer annealing temperatures used for genotyping and for sequence analysis are given in the supplementary data section (Supplemental Table S1 and S2). The results of the genotyping for the CD-associated *ATG16L1* variant rs2241880 (p.Thr300Ala) were available from a previous study [29].

### Statistical analyses

Each genetic marker was tested for Hardy-Weinberg equilibrium in the control population. Fisher's exact test or χ^2^ test for comparison between categorical variables were used where appropriate. Single-marker allelic tests were performed with Pearson's χ^2^ test. Student's t-test was applied for quantitative variables. All tests were two-tailed and p-values <0.05 were considered significant. Odds ratios were calculated for the minor allele at each SNP. For evaluation of phenotypic consequences, we conducted a logistic regression analysis. Data were evaluated by using the SPSS 13.0 software (SPSS Inc., Chicago, IL, U.S.A.) and R-2.4.1. (http://cran.r-project.org). Haplotype analysis and calculation of linkage disequilibrium (LD) were conducted using PLINK (http://pngu.mgh.harvard.edu/~purcell/plink/).

## Results

### The novel NOD2 variants rs2066843 and rs2076756 are associated with susceptibility to CD but not to UC

In the controls, the genotype frequencies of the SNPs rs2066843 and rs2076756 were in agreement with the predicted Hardy-Weinberg equilibrium. Significant differences in the allele frequencies of the SNPs rs2066843 and rs2076756 were observed in CD patients but not in UC patients compared to healthy controls. As summarized in Table 2, the SNPs rs2066843 and rs2076756 were strongly associated with CD. In the group of CD patients, the frequency of the rarer T allele of the rs2066843 variant was 0.390, whereas in the controls it was 0.299 (p = 3.01×10^−5^; OR 1.48, [95% CI 1.23-1.78]). The frequencies of the less common G allele of the rs2076756 variant were 0.390 in CD and 0.286 in the controls (p = 4.01×10^−6^; OR 1.54, [95% CI 1.28-1.86]). In contrast to CD, no associations of both *NOD2* SNPs were observed in UC. The frequency of the rs2066843 T allele was 0.300 (p = 9.67×10^−1^, OR 1.01 [95% CI 0.85-1.19]), while the frequency of the rs2076756 G allele was 0.270 (p = 3.74×10^−1^, OR 1.09 [95% CI 0.94-1.27]). The association of the *NOD2* SNPs with CD susceptibility was found in both our initial discovery cohort from the University Hospital Munich-Grosshadern (Supplemental Table S3) and the replication cohort from the University Hospital Munich, Campus Innenstadt and from Ruhr-University Bochum (Supplemental Table S4)**.** In this study, rs2066843 and rs2076756 were in LD in all studied subpopulations (CD, UC, healthy controls; Supplemental Tables S5, S6, S7).

**Table 2:** Allele frequencies of the SNPs rs2066843 and rs2076756 in patients with Crohn's disease, ulcerative colitis and controls.

Gene marker	Minor allele	Crohn's diseasen = 812			Ulcerative colitisn = 442			Controlsn = 1446
		MAF	p value	OR [95% CI]	MAF	p value	OR [95% CI]	MAF
rs2066843	T	0.390	3.01×10^−5^	1.48 [1.23–1.78]	0.300	9.67×10^−1^	1.01 [0.85–1.19]	0.299
rs2076756	G	0.380	4.01×10^−6^	1.54 [1.28–1.86]	0.270	3.74×10^−1^	1.09 [0.94–1.27]	0.286
rs2066844 p.Arg702Trp	T	0.089	1.43×10^−6^	2.07 [1.53–2.79]	0.046	7.49×10^−1^	0.92 [0.60–1.40]	0.050
rs2066845 p.Gly908Arg	G	0.042	1.1×10^−2^	1.72 [1.14–2.60]	0.022	1.00	0.94 [0.51–1.73]	0.024
rs2066847 p.Leu1007fsX1008	insC	0.121	1.88×10^−14^	5.03 [3.54–7.15]	0.022	3.91×10^−1^	0.76 [0.42–1.37]	0.028

Minor allele frequencies (MAF), allelic test *P*-values, and odds ratios (OR, shown for the minor allele) with 95% confidence intervals (CI) are depicted for both the CD and UC case-control cohorts.

To analyze for potential disease associations with certain NOD2 haplotypes, we performed a detailed haplotype analysis (Table 3 and Supplemental Table S8). As shown in Table 3, we demonstrated for a number of haplotypes significant associations with CD susceptibility, including several associations with omnibus p values of less than 10^−10^. The strongest association of a haplotype including one of the SNPs rs2066843 or rs2076756 comprised of the NOD2 SNPs rs2066844-rs2066845-rs2066847-rs2076756 (omnibus p-value  = 1.14×10^−23^). In contrast, no significant associations were found with UC susceptibility (Supplemental Table S8).

**Table 3:** Haplotype analysis for *NOD2* SNPs in the CD patient cohort.

*NOD2* haplotypes	p-value	OR	CI lower	CI upper
**rs2066843-rs2066844**	1.31×10^−8^			
TT	1.34×10^−5^	1.94	1.81	2.08
TC	3.09×10^−4^	1.34	1.28	1.40
CC	1.72×10^−8^	0.65	0.63	0.66
**rs2066844-rs2066845**	7.94×10^−8^			
CC	3.25×10^−3^	1.80	1.58	2.06
TG	3.80×10^−6^	1.89	1.78	2.00
CG	1.08×10^−8^	0.52	0.50	0.54
**rs2066845-rs2066847**	1.09×10^−18^			
GC	6.88×10^−17^	4.06	3.90	4.22
CX	6.67×10^−3^	1.74	1.50	2.02
GX	4.38×10^−20^	0.32	0.31	0.33
**rs2066847-rs2076756**	2.48×10^−15^			
CG	2.88×10^−14^	3.84	3.67	4.02
XG	3.31×10^−1^	1.09	0.91	1.30
XA	2.35×10^−9^	0.62	0.61	0.64
**rs2066843-rs2066844-rs2066845**	5.94×10^−10^			
TCC	1.16×10^−3^	1.90	1.69	2.14
TTG	8.40×10^−7^	2.01	1.90	2.13
TCG	2.64×10^−2^	1.21	1.12	1.31
CCG	9.68×10^−10^	0.62	0.61	0.64
**rs2066844-rs2066845-rs2066847**	8.22×10^−24^			
CGC	5.84×10^−17^	4.07	3.91	4.23
CCX	1.08×10^−2^	1.70	1.45	1.99
TGX	3.59×10^−6^	1.89	1.78	2.00
CGX	4.19×10^−26^	0.37	0.36	0.37
**rs2066845-rs2066847-rs2076756**	2.48×10^−15^			
GCG	5.41×10^−17^	4.09	3.93	4.25
CXG	1.04×10^−3^	1.94	1.72	2.19
GXG	3.47×10^−1^	0.92	0.76	1.11
GXA	3.89×10^−12^	0.59	0.57	0.60
**rs2066843-rs2066844-rs2066845-rs2066847**	1.60×10^−23^			
TCGC	4.10×10^−17^	4.28	4.11	4.46
TCCX	1.18×10^−3^	1.90	1.69	2.14
TTGX	8.51×10^−7^	1.99	1.88	2.10
TCGX	2.32×10^−5^	0.66	0.63	0.69
CCGX	8.57×10^−13^	0.59	0.58	0.60
**rs2066844-rs2066845-rs2066847-rs2076756**	1.14×10^−23^			
CGCG	5.20×10^−17^	4.04	3.89	4.20
CCXG	1.10×10^−3^	1.93	1.71	2.18
TGXG	4.75×10^−7^	2.06	1.95	2.18
CGXG	1.24×10^−5^	0.642	0.61	0.67
CGXA	7.82×10^−13^	0.59		
**rs2066843-rs2066844-rs2066845-rs2066847-rs2076756**	1.43×10^−21^			
TCGCG	6.33×10^−17^	4.18	4.02	4.35
TCCXG	1.07×10^−3^	1.93	1.71	2.18
TTGXG	6.00×10^−7^	2.05	1.94	2.17
TCGXG	9.42×10^−5^	0.67	0.64	0.71
TCGXA	6.48×10^−2^	0.576	0.42	0.79
CCGXA	2.01×10^−11^	0.61	0.60	0.63

Omnibus as well as individual p-values are presented.

### Genotype-phenotype analysis of rs2066843 and rs2076756 NOD2 variants in CD patients

Since the three common *NOD2* variants p.Arg702Trp (rs2066844), p.Gly908Arg (rs2066847), and p.Leu1007fsX1008 (rs2066847) have been identified to be associated with a certain CD phenotype [21], [22], [23], [24], [30], we also performed a detailed genotype-phenotype correlation in IBD patients. In univariate analysis, CD patients homozygous for the SNP rs2066843 were found to have a lower body mass index (p = 0.039) and less colonic involvement (p = 0.041) compared to the wildtype patients and we observed also a trend towards a predominantly penetrating disease phenotype (B3) (p = 0.068; Table 4). In addition, a higher need for CD-related surgery in homozygous carriers of the SNP rs2066843 (p = 0.015) was observed compared to the wildtype group (Table 5).

**Table 4:** Association between rs2066843 genotype and CD disease characteristics based on the Montreal classification [28].

rs2066843 genotype status	(1)CCn = 322	(2)CTn = 345	(3)TTn = 150	(1) vs. (2)p-valueOR [95% CI]	(1) vs. (3)p-valueOR [95% CI]	(1) vs. (2) + (3)p-valueOR [95% CI]
**Age at diagnosis**≤16 years (A1)17–40 years (A2)>40 years (A3)	74/239 (31.0%)150/239 (62.8%)15/239 (6.2%)	81/237 (34.2%)125/237 (52.7%)31/237 (13.1%)	37/116 (31.9%)69/116 (59.5%)10/116 (8.6%)	0.4941.16 (0.79–1.70)**0.033**0.66 (0.46–0.95)**0.013**2.25 (1.18–4.28)	0.9031.04 (0.65–1.68)0.5620.87 (0.55–1.37)0.5071.41 (0.61–3.24)	0.5911.12 (0.79–1.59)0.0620.72 (0.52–1.01)**0.032**1.96 (1.06–3.63)
**Location**Terminal ileum (L1)Colon (L2)Ileocolon (L3)Upper GI (L4)Any ileal involvement(L1+L3)	32/244 (13.1%)42/244 (17.2%)166/244 (67.9%)4/244 (1.6%)198/244 (81.1%)	46/238 (19.3%)33/238 (13.9%)155/238 (65.1%)4/238 (1.7%)201/238 (84.5%)	12/120 (10.0%)11/120 (9.2%)93/120 (77.5%)4/120 (3.3%)105/120 (87.5%)	0.0831.59 (0.97–2.59)0.3180.77 (0.47–1.27)0.5010.88 (0.60–1.28)1.0001.03 (0.25–4.15)0.3981.26 (0.78–2.03)	0.4940.74 (0.36–1.49)**0.041**0.48 (0.24–0.98)0.0661.62 (0.98–2.68)0.4472.07 (0.51–8.42)0.1381.63 (0.87–3.05)	0.3521.28 (0.80–2.04)0.0970.67 (0.43–1.07)0.7881.06 (0.75–1.50)0.7701.37 (0.41–4.61)0.1771.37 (0.88–2.11)
**Behaviour** 1Non-stricturing,Non-penetrat. (B1)Stricturing (B2)Penetrating (B3)	49/232 (21.1%)66/232 (28.4%)117/232 (50.4%)	61/233 (26.2%)64/233 (27.5%)108/233 (46.3%)	18/116 (15.5%)27/116 (23.3%)71/116 (61.2%)	0.2301.32 (0.86–2.04)0.8370.95 (0.63–1.43)0.4040.85 (0.59–1.22)	0.2490.69 (0.38–1.24)0.3680.76 (0.45–1.28)0.0681.55 (0.98–2.44)	0.6841.09 (0.73–1.63)0.5670.89 (0.61–1.29)0.8661.03 (0.74–1.44)

1 Disease behaviour was defined according to the Montreal classification [28]. Stricturing disease phenotype was defined as presence of stenosis without penetrating disease. The diagnosis of stenosis was made surgically, endoscopically, or radiologically (using MR enteroclysis). For each variable, the number of patients included is given.

**Table 5:** Association between rs2066843 genotype and CD disease characteristics.

rs2066843 genotype status	(1)CCn = 322	(2)CTn = 345	(3)TTn = 150	(1) vs. (2)p-valueOR [95% CI]	(1) vs. (3)p-valueOR [95% CI]	(1) vs. (2) + (3)p-valueOR [95% CI]
**Male sex**	129/277 (46.6%)	153/289 (52.9%)	74/134 (55.2%)	0.1311.29 (0.93–1.80)	0.1151.41 (0.94–2.14)	0.0751.33 (0.98–1.80)
**Age at diagnosis (yrs)**Mean ± SDRange	27.1±10.911–70	28.7±12.51–78	25.8±11.36–71	0.151	0.302	0.526
**Disease duration (yrs)**Mean ± SDRange	12.3±8.40–37	11.4±9.011–44	11.8±8.21–35	0.381	0.675	0.403
**Body mass index (yrs)**Mean ± SDRange	23.3±4.416–40	23.4±4.016–37	22.0±3.613–31	0.845	**0.039**	0.473
**Use of immunosuppressive agents** 2	141/169 (83.4%)	135/175 (77.1%)	70/83 (84.3%)	0.1760.67 (0.39–1.15)	1.0001.07 (0.52–2.19)	0.3160.77 (0.46–1.27)
**Surgery because of CD** 3	113/221 (51.1%)	120/224 (53.6%)	76/116 (65.5%)	0.6361.10 (0.76–1.60)	**0.015**1.82 (1.14–2.89)	0.1401.30 (0.93–1.83)
**Fistulas**	117/232 (50.4%)	108/233 (46.3%)	71/116 (61.2%)	0.4040.85 (0.59–1.22)	0.0681.55 (0.98–2.44)	0.8661.03 (0.74–1.44)
**Perianal fistulas**	24/92 (26.1%)	18/90 (20.0%)	7/49 (14.3%)	0.2121.41 (0.70–2.83)	0.0792.12 (0.84–5.34)	0.0961.61 (0.85–3.04)
**Stenosis**	140/229 (61.1%)	144/233 (61.8%)	80/115 (69.6%)	0.9241.03 (0.71–1.50)	0.1531.45 (0.90–2.34)	0.4811.15 (0.81–1.62)

2 Immunosuppressive agents included azathioprine, 6-mercaptopurine, 6-thioguanine, methotrexate, and/or infliximab.

3 Only surgery related to CD-specific problems (e.g. fistulectomy, colectomy, ileostomy) was included. For each variable, the number of patients included is given.

Analyzing CD patients regarding the rs2076756 genotype status, a significant younger age at disease onset was observed in homozygous carriers (mean 25.8±11.4 years) compared to wildtype patients (p = 0.023; Table 6). Similar to the analysis of SNP rs2066843, homozygous carriers of SNP rs2076756 had less colonic involvement than wildtype patients (p = 0.032) but showed a trend towards ileocolonic disease location (p = 0.058) and had a significant higher rate of penetrating disease phenotype (B3) (p = 0.015; Table 6). The significant association of SNP rs2076756 with a severe disease phenotype was also reflected by the significantly higher percentage of patients with CD-related surgery (p = 0.003) and internal fistulas (p = 0.015) in homozygous carriers of this SNP (Table 7). Moreover, there was also a trend towards stenotic complications (p = 0.067) in homozygous CD patients (Table 7). However, given the large number of associations investigated, most associations lost significance following Bonferroni correction.

**Table 6:** Association between rs2076756 genotype and CD disease characteristics based on the Montreal classification [28].

rs2076756 genotype status	(1)AAn = 339	(2)AGn = 334	(3)GGn = 143	(1) vs. (2)p-valueOR [95% CI]	(1) vs. (3)p-valueOR [95% CI]	(1) vs. (2) + (3)p-valueOR [95% CI]
**Age at diagnosis**≤16 years (A1)17–40 years (A2)>40 years (A3)	73/241(30.3%)154/241(63.9%)14/241 (5.8%)	75/233 (32.2%)124/233 (53.2%)34/233 (14.6%)	35/110 (31.8%)66/110 (60.0%)9/110 (8.2%)	0.6921.09 (0.74–1.61)**0.020**0.64 (0.44–0.93)**0.002**2.77 (1.44–5.31)	0.8041.07 (0.66–1.75)0.5520.85 (0.53–1.35)0.4861.44 (0.61–3.45)	0.7171.09 (0.76–1.55)**0.041**0.70 (0.50–0.98)**0.007**2.32 (1.24–4.35)
**Location**Terminal ileum (L1)Colon (L2)Ileocolon (L3)Upper GI (L4)IleocolonicInvolvement	31/245 (12.7%)41/245 (16.7%)169/245 (70.0%)4/245 (1.6%)200/245 (81.6%)	45/235 (19.1%)34/235 (14.5%)152/235 (64.7%)4/235 (1.7%)197/235 (83.8%)	11/113 (9.7%)9/113 (8.0%)89/113 (78.8%)4/113 (3.5%)100/113 (88.5%)	0.0611.63 (0.99–2.69)0.5310.84 (0.51–1.38)0.3330.82 (0.56–1.20)1.0001.04 (0.26–4.22)0.5481.17 (0.73–1.87)	0.4830.74 (0.36–1.54)**0.032**0.43 (0.20–0.92)0.0581.67 (0.99–2.82)0.2682.21 (0.54–9.00)0.1231.73 (0.89–3.36)	0.2891.32 (0.82–2.12)0.1510.70 (0.44–1.11)1.0001.01 (0.71–1.44)0.7691.48 (0.42–4.76)0.2581.31 (0.84–2.03)
**Behaviour** ^1^Non-stricturing,Non-penetrat. (B1)Stricturing (B2)Penetrating (B3)	49/232 (21.1%)67/232 (28.9%)116/232 (50.0%)	64/231 (27.7%961/231 (26.4%)106/231 (45.9%)	12/109 (11.0%)27/109 (24.8%)70/109 (64.2%)	0.1061.43 (0.93–2.19)0.6040.88 (0.59–1.33)0.4030.85 (0.59–1.22)	0.0230.46 (0.23–0.91)0.5160.81 (0.48–1.36)0.0151.79 (1.12–2.87)	0.7581.07 (0.72–1.61)0.4440.86 (0.59–1.25)0.7331.07 (0.77–1.50)

**Table 7:** Association between rs2076756 genotype and CD disease characteristics.

rs2076756 genotype status	(1)AAn = 339	(2)AGn = 334	(3)GGn = 143	(1) vs. (2)p-valueOR [95% CI]	(1) vs. (3)p-valueOR [95% CI]	(1) vs. (2) + (3)p-valueOR [95% CI]
**Male sex**	129/280 (46.1%)	152/283 (53.7%)	69/127 (54.3%)	0.0771.36 (0.75–1.89)	0.1351.39 (0.91–2.12)	**0.044**1.37 (1.01–1.86)
**Age at diagnosis (yr)**Mean ± SDRange	26.8±10.511–70	29.2±12.71–78	25.8±11.46–71	**0.023**	0.465	0.157
**Disease duration (yr)**Mean ± SDRange	12.4±8.40–37	11.2±8.81–44	11.6±8.21–35	0.188	0.507	0.202
**Body mass index (yr)**Mean ± SDRange	23.4±10.511–70	23.2±4.016–37	22.3±3.713–31	0.691	0.074	0.309
**Use of immunosuppressive agents** ^2^	147/174 (84.5%)	136/176 (77.3%)	65/80 (81.2%)	0.1030.62 (0.36–1.07)	0.5860.80 (0.40–1.59)	0.1340.67 (0.40–1.11)
**Surgery because of CD** ^3^	114/221 (51.6%)	120/222 (54.1%)	76/110 (69.1%)	0.6351.10 (0.76–1.60)	**0.003**2.10 (1.29–3.40)	0.0971.35 (0.96–1.91)
**Fistulas**	116/232 (50.0%)	106/231 (45.9%)	70/109 (64.2%)	0.4030.85 (0.59–1.22)	**0.015**1.79 (1.12–2.87)	0.7331.07 (0.77–1.50)
**Perianal fistulas**	21/95 (22.1%)	21/90 (23.3%)	5/47 (10.6%)	0.6460.93 (0.47–1.86)	0.0732.38 (0.84–6.79)	0.3371.21 (0.64–2.31)
**Stenosis**	142/229 (62.0%)	139/230 (60.4%)	79/109 (72.5%)	0.7740.94 (0.64–1.36)	0.0671.61 (0.98–2.65)	0.5951.10 (0.78–1.56)

For each variable, the number of patients included is given. Footnotes: see Table 4 and 5 for details.

Given the increased prevalence of the three common *NOD2* variants among carriers of the risk allele of rs2066843 and rs2076756 and previous reports demonstrating a severe phenotype in carriers of these three *NOD2* variants, we next investigated if the phenotypic effects of rs2066843 and rs2076756 were independent of the main three CD-associated *NOD2* variants p.Arg702Trp (rs2066844), p.Gly908Arg (rs2066847), and p.Leu1007fsX1008 (rs2066847). As shown in Table 8, there were no significant differences regarding homozygous carriers of these SNPs when stratified for the presence and absence of the main three CD-associated *NOD2* variants and the significant phenotypic characteristics found in Tables 5, 6 and 7, suggesting that the CD-modifying effect of rs2066843 and rs2076756 is independent of the three main *NOD2* variants.

**Table 8:** Significant CD phenotype associations of rs2066843 and rs2076756 as shown in Tables 5, 6 and 7 stratified for the presence (*NOD2+*) or absence (*NOD2-*) of the three CD-associated *NOD2* mutations p.Arg702Trp (rs2066844), p.Gly908Arg (rs2066847), and p.Leu1007fsX1008 (rs2066847).

	*NOD2+*n (%)	*NOD2-*n (%)	p value
**rs2066843-TT carriers**			
** Surgery because of CD**	65/95 (68.4%)	11/21 (52.4%)	0.206
	***NOD2+*** **n (%)**	***NOD2-*** **n (%)**	
**rs2076756-GG carriers**			
** Surgery because of CD**	67/96 (69.8%)	9/14 (64.3%)	0.759
** B3**	63/96 (65.6%)	8/13 (61.5%)	0.765
** Fistulas**	63/96 (65.6%)	8/13 (61.5%)	0.765

Analyzing potential therapeutic consequences such as need for surgery, we next conducted a logistic regression analysis with R, using the need for surgery as dependent variable, and the SNP genotype as independent variable, taking localization as well as behaviour as covariates. This revealed that disease localization has a significant influence on the need for surgery (p = 0.02). In addition, disease behaviour is significantly associated with the need for surgery (p = 2.0×10^−16^, independently of localization). However, using the *NOD2* genotype status as further explanatory variable does not improve the model fit (F-test p = 0.36 rs2066843, p = 0.32 rs2076756).

In UC patients, the analysis revealed no significant associations of the SNPs rs2066843 and rs2076756 with phenotypic characteristics such as age, age a diagnosis, male-to-female-ratio, body mass index (BMI), family history, anatomic location and disease behaviour, use of immunosuppressive agents, or UC-related complications (data not shown).

### No evidence for epistasis between NOD2 variants and the CD-associated ATG16L1 variant rs2241880 (p.Thr300Ala)

Very recent studies indicate that NOD2 recruits the autophagy protein ATG16L1 to the plasma membrane at the bacterial entry site [31]. In contrast, CD-associated mutants failed to recruit ATG16L1 to the plasma membrane and wrapping of invading bacteria by autophagosomes was impaired [31]. Moreover, dendritic cells from CD patients expressing CD-associated *NOD2* or *ATG16L1* risk variants are defective in autophagy induction, bacterial trafficking and antigen presentation [32]. We therefore hypothesized that there may be epistasis between CD-associated *NOD2* and *ATG16L1* variants regarding susceptibility to CD. However, as shown in Supplemental Table S9, none of the 5 CD-associated *NOD2* variants showed evidence for epistasis to the CD-associated *ATG16L1* variant rs2241880 (p.Thr300Ala).

## Discussion

The identification of *NOD2* as the first CD susceptibility gene in 2001 represents a landmark finding that implicated bacterial recognition and innate immunity as key processes involved in the pathogenesis of CD. Since genotype-phenotype analyses of the *NOD2* variants p.Arg702Trp, p.Gly908Arg, and p.Leu1007fsX1008 have also provided strong evidence for the existence of a *NOD2-*related CD phenotype, these studies have not only changed our understanding of IBD pathogenesis but have also implications for clinical practice [21], [22], [23], [25].

Here, we investigated the two novel CD-associated *NOD2* variants rs2066843 and rs2076756 in a large German IBD patient cohort, confirming these *NOD2* variants as susceptibility gene variants for CD but not for UC. We could demonstrate a highly significant association of SNPs rs2066843 and rs2076756 with CD. The haplotype analysis revealed a number of significant associations with CD susceptibility, including several associations with omnibus p values of less than 10^−10^.

In addition, for the first time, our genotype-phenotype analysis revealed a significant association of the SNPs rs2066843 and rs2076756 with phenotypic characteristics such as early disease onset, severe penetrating disease phenotype complicated by fistulas. Moreover, univariante analysis revealed a frequent need for CD-related surgery associated with SNPs rs2066843 and rs2076756 in CD patients. However, the two novel CD-associated NOD2 variants could not be identified as independent variables for the need for surgery in CD patients after logistic regression analysis, suggesting that ileal disease localization and a stricturing or penetrating phenotype are clinically more relevant predictors for the need for CD-related surgery than the NOD2 genotypes alone. In addition, the strength of the association of rs2066843 and rs2076756 with CD was less pronounced than that of the NOD2 variant rs2066847 (p.Leu1007fsX1008) which results in patients homozygous for this variant in a more severe phenotype [21], [22] than found for rs2066843 and rs2076756 in this study. Early deep-sequencing studies of the NOD2 locus by the Hugot group also suggested that the NOD2 “gene-dosage” effect is more important in the CD phenotype development than the type of NOD2 mutation ^25^. In the study by the Hugot group, patients with “double-dose” NOD2 mutations (homozygous or compound heterozygous) were characterized by a younger age at onset, a more frequent stricturing phenotype, and less frequent colonic involvement than were seen in those patients who had no mutation ^25^.

Although the phenotypic effects of rs2066843 and rs2076756 were independent of the presence of the three common *NOD2* variants p.Arg702Trp, p.Gly908Arg, and p.Leu1007fsX1008, the observed associations demonstrate similarities to the phenotype as previously shown to be related to the three common *NOD2* variants in our cohort [21], [22]. The explorative character of our study has to be acknowledged. Given the large number of associations investigated, most associations would loose significance following Bonferroni correction. However, considering the size of our cohort and given that the highest percentages of carriers with ileocolonic involvement, stenoses, fistulas, and CD-related surgery were found without exception among the homozygous carriers of these two novel *NOD2* SNPs, an association with a severe CD phenotype is very likely. Moreover, this result has to be seen in the background of a study population of a tertiary referral center with a very high percentage of severe CD demonstrated by fistulas and stenoses and CD-related surgery in more than half of the study population regardless of the genotype. These phenotypic associations found in homozygous carriers of these SNPs were demonstrated with similar frequencies in the subgroup of CD patients with additional CD-associated *NOD2* mutations and in patients without these mutations, suggesting a specific disease-modifying effect of rs2066843 and rs2076756.

Our findings highlight the essential role of the *NOD2* region as CD susceptibility gene encoding a protein which acts as sensor for bacterial muramyl dipeptide (MDP) in the cell wall of Gram-positive and Gram-negative bacteria [8], [15], [16], [17]. However, the exact mechanism how *NOD2* variants influence the intestinal inflammation is still under investigation. First, *NOD2* mutations are associated with diminished mucosal alpha-defensin expression in CD [33], although this finding is challenged by the results of a recent study [34], demonstrating that in ileal CD reduced alpha-defensin expression is the result of inflammation and not of *NOD2* mutation status. Second, there is evidence for an impaired dendritic cell function in CD patients with *NOD2* variants [35] and a loss of synergy between TLR9 and NOD2 in innate immune responses to CpG DNA [36]. Third, there might be cross-tolerization between NOD1 and NOD2 leading to increased recognition of both pathogenic and commensal bacteria in NOD2-deficient macrophages pre-exposed to microbial ligands [37]. Fourth, the study by Kobayashi et al. demontrated that *Nod2*-deficient mice are more susceptible to bacterial infection and thus Nod2 protein is a critical regulator of bacterial immunity within the intestine [38]. Finally, very recent evidence suggests that NOD1 and NOD2 are essential for recruitment of ATG16L1 during bacterial infection [31], which is impaired in patients with CD-associated *NOD* mutants [31]. We therefore analyzed CD-associated *NOD2* and *ATG16L1* variants for epistasis. However, we found no evidence for significant gene-gene interactions between these variants regarding CD susceptibility. This is in line with another large study demonstrating no epistasis for the three main CD-associated *NOD2* variants and *ATG16L1* [39], while another study found a weak gene-gene interaction between the *ATG16L1* variant rs2241880 and the three common CD-associated *NOD2* variants (p = 0.039) [14].

Despite the growing evidence for a strong genetic background in IBD by various genome-wide analyses and cohort studies [7], [11], [13], [14], [40], [41], [42], [43], [44], CD still remains a complex disorder modulated not only by susceptibility genes but also influenced by environmental factors. Moreover, there are genetic differences between different ethnic cohorts. For example, studies in Asian CD cohorts [45], [46] could not show any evidence for a role of *NOD2* in CD susceptibility in Asian populations. Genetic counselling based on the *NOD2* genotype as well as the analysis of environmental risk factors could therefore benefit the individual at risk and change daily clinical practice.

Taken together, we conclude that the identification of the two novel *NOD2* variants rs2066843 and rs2076756 might have implications for the future risk assessment in CD patients, considering their high minor allele frequencies among Caucasian CD patients and the association with a severe disease phenotype. However, the association of these two *NOD2* variants with CD was less pronounced than that with rs2066847 (p.Leu1007fsX1008) which demonstrated even a more severe phenotypic effect than rs2066843 and rs2076756 in our previous studies [21], [22]. So far, a major functional effect on the NF-κB pathway has only be shown for the p.Leu1007fsX1008 variant ^6^. Therefore, further functional analyses of rs2066843 and rs2076756 as well as detailed genotype-phenotype analyses in very large cohorts including a comprehensive assessment of low frequency variants in the *NOD2* gene region are needed to clarify the contribution of these novel variants to the pathogenesis of CD. Currently, these two novel variants will not replace the other common CD-associated *NOD2* variants p.Arg702Trp (rs2066844), p.Gly908Arg (rs2066847), and p.Leu1007fsX1008 (rs2066847) when evaluating genetic risk factors in CD patients.

## Supporting Information

Table S1 Primer sequences, FRET probe sequences and primer annealing temperatures used for genotyping of NOD2 variants rs2066843 and rs2076756. Note: FL: Fluorescein, LC640: LightCycler Red 640, LC670: LightCycler Red 670; the polymorphic position within the sensor probe is underlined. A phosphate is linked to the 3′-end of the acceptor probe to prevent elongation by the DNA polymerase in the PCR.
(0.02 MB DOC)

Table S2 Primer sequences used for sequence analysis of NOD2 variants rs2066843 and rs2076756.
(0.02 MB DOC)

Table S3 Allele frequencies of the SNPs rs2066843 and rs2076756 in patients with Crohn's disease (CD), ulcerative colitis (UC) and controls in the initial discovery cohort from the University Hospital Munich-Grosshadern. Minor allele frequencies (MAF), allelic test P-values, and odds ratios (OR, shown for the minor allele) with 95% confidence intervals (CI) are depicted for both the CD and UC case-control cohorts.
(0.03 MB DOC)

Table S4 Allele frequencies of the NOD2 SNPs in patients with Crohn's disease (CD), ulcerative colitis (UC) and controls from the replication cohort of the University Hospital Munich, Campus Innenstadt and Ruhr-University Bochum. Minor allele frequencies (MAF), allelic test P-values, and odds ratios (OR, shown for the minor allele) with 95% confidence intervals (CI) are depicted for both the CD and UC case-control cohorts.
(0.03 MB DOC)

Table S5 LD matrix for NOD2 SNPs in CD patients. Values are given as D'/r^2^.
(0.02 MB DOC)

Table S6 LD matrix for NOD2 SNPs in UC patients. Values are given as D'/r^2^.
(0.03 MB DOC)

Table S7 LD matrix for NOD2 SNPs in controls. Values are given as D'/r^2^.
(0.03 MB DOC)

Table S8 Haplotype-analysis for NOD2 SNPs in the UC patient cohort. Only omnibus p-values are presented, given that none of these haplotypes showed significant disease association.
(0.02 MB DOC)

Table S9 Analysis for epistasis between SNPs rs2066843, rs2066843, rs2066844 (p.Arg702Trp), rs2066845 (p.Gly908Arg) and rs2066847 (p.Leu1007fsX1008) in the NOD2 gene and the SNP rs2241880 = p.Thr300Ala within the ATG16L1 gene regarding CD susceptibility. *All p values given are uncorrected for multiple comparisons.
(0.02 MB DOC)
