# Supplementary material for: The NOD2 Single Nucleotide Polymorphisms rs2066843 and rs2076756 Are Novel and Common Crohn's Disease Susceptibility Gene Variants
Source: PLoS One. 2010 Dec 30;5(12):e14466. doi: 10.1371/journal.pone.0014466 (PMC3012690; doi:10.1371/journal.pone.0014466)
Supplement: Table S1 — Primer sequences, FRET probe sequences and primer annealing temperatures used for genotyping of NOD2 variants rs2066843 and rs2076756. Note: FL: Fluorescein, LC640: LightCycler Red 640, LC670: LightCycler Red 670; the polymorphic position within the sensor probe is underlined. A phosphate is linked to the 3′-end of the acceptor probe to prevent elongation by the DNA polymerase in the PCR. (0.02 MB DOC) [file pone.0014466.s001.doc]

# Supplementary data

**Supplemental Table S1.**

| **Polymorphism** | **Primer sequences** | **Primer annealing** | **FRET probe sequences** |
| --- | --- | --- | --- |
| rs2066843 | GAAGTACATCCGCACCGAGT  AACAGTTCCTGGTGGCATT | 60 °C | ATGATGACGCTTCCTCAGGT-FL  LC640-CAGCTCGATGCCCTGTTCAGAGAAGCCC |
| rs2076756 | GCTCTCCTGGTCTGCTCC  GAAATGTCCCTTGTCCTCTCA | 60 °C | LC670-TTGGAATTGGCCCTTAAGATA  TGCACTGGGCACCCACTACCAATGG-FL |
