# Supplementary material for: The NOD2 Single Nucleotide Polymorphisms rs2066843 and rs2076756 Are Novel and Common Crohn's Disease Susceptibility Gene Variants
Source: PLoS One. 2010 Dec 30;5(12):e14466. doi: 10.1371/journal.pone.0014466 (PMC3012690; doi:10.1371/journal.pone.0014466)
Supplement: Table S2 — Primer sequences used for sequence analysis of NOD2 variants rs2066843 and rs2076756. (0.02 MB DOC) [file pone.0014466.s002.doc]

**Supplemental Table S2.**

| ***NOD2* Polymorphism** | **Primer sequences** |
| --- | --- |
| rs2066843 | GAAGTACATCCGCACCGAGT  GCAGGTACATATCTGTAGTGGTCTTT |
| rs2076756 | ACCTCAGAGATGAGCTAACCTATG  GAAATGTCCCTTGTCCTCTCA |
