# Supplementary material for: The NOD2 Single Nucleotide Polymorphisms rs2066843 and rs2076756 Are Novel and Common Crohn's Disease Susceptibility Gene Variants
Source: PLoS One. 2010 Dec 30;5(12):e14466. doi: 10.1371/journal.pone.0014466 (PMC3012690; doi:10.1371/journal.pone.0014466)
Supplement: Table S3 — Allele frequencies of the SNPs rs2066843 and rs2076756 in patients with Crohn's disease (CD), ulcerative colitis (UC) and controls in the initial discovery cohort from the University Hospital Munich-Grosshadern. Minor allele frequencies (MAF), allelic test P-values, and odds ratios (OR, shown for the minor allele) with 95% confidence intervals (CI) are depicted for both the CD and UC case-control cohorts. (0.03 MB DOC) [file pone.0014466.s003.doc]

**Supplemental Table S3.**

| **Gene marker** | **Minor allele** | **Crohn’s disease**  n=519 | | | **Ulcerative colitis**  n=232 | | | **Controls**  n=770 |
| --- | --- | --- | --- | --- | --- | --- | --- | --- |
|  |  | **MAF** | **p value** | **OR [95 % CI]** | **MAF** | **p value** | **OR [95 % CI]** | **MAF** |
| rs2066843 | T | 0.38 | 4.33 x 10^-7^ | 1.54 [1.30-1.82] | 0.28 | 0.462 | 0.91 [0.70-1.16] | 0.30 |
| rs2076756 | G | 0.37 | 1.03 x 10^-7^ | 1.57 [1.33-1.87] | 0.27 | 0.681 | 0.95 [0.74-1.20] | 0.28 |
