# Supplementary material for: The NOD2 Single Nucleotide Polymorphisms rs2066843 and rs2076756 Are Novel and Common Crohn's Disease Susceptibility Gene Variants
Source: PLoS One. 2010 Dec 30;5(12):e14466. doi: 10.1371/journal.pone.0014466 (PMC3012690; doi:10.1371/journal.pone.0014466)
Supplement: Table S4 — Allele frequencies of the NOD2 SNPs in patients with Crohn's disease (CD), ulcerative colitis (UC) and controls from the replication cohort of the University Hospital Munich, Campus Innenstadt and Ruhr-University Bochum. Minor allele frequencies (MAF), allelic test P-values, and odds ratios (OR, shown for the minor allele) with 95% confidence intervals (CI) are depicted for both the CD and UC case-control cohorts. (0.03 MB DOC) [file pone.0014466.s004.doc]

**Supplemental Table S4.**

| **Gene marker** | **Minor allele** | **Crohn’s disease**  n=293 | | | **Ulcerative colitis**  n=210 | | | **Controls**  n=676 |
| --- | --- | --- | --- | --- | --- | --- | --- | --- |
|  |  | **MAF** | **p value** | **OR [95 % CI]** | **MAF** | **p value** | **OR [95 % CI]** | **MAF** |
| rs2066843 | T | 0.40 | 8.50 x 10^-4^ | 1.42 [1.15-1.75] | 0.32 | 0.576 | 0.92 [0.71-1.10] | 0.30 |
| rs2076756 | G | 0.39 | 5.01 x 10^-4^ | 1.44 [1.17-1.78] | 0.27 | 1.000 | 0.99 [0.77-1.28] | 0.29 |
