# Supplementary material for: The NOD2 Single Nucleotide Polymorphisms rs2066843 and rs2076756 Are Novel and Common Crohn's Disease Susceptibility Gene Variants
Source: PLoS One. 2010 Dec 30;5(12):e14466. doi: 10.1371/journal.pone.0014466 (PMC3012690; doi:10.1371/journal.pone.0014466)
Supplement: Table S5 — LD matrix for NOD2 SNPs in CD patients. Values are given as D'/r2. (0.02 MB DOC) [file pone.0014466.s005.doc]

**Supplemental Table S5.**

| ***NOD2* SNPs** | **rs2066843** | **rs2066844** | **rs2066845** | **rs2066847** | **rs2076756** |
| --- | --- | --- | --- | --- | --- |
| **rs2066843** | * | 0.96/0.14 | 1.00/0.07 | 0.90/0.18 | 0.97/0.90 |
| **rs2066844** | * | * | 0.47/0.001 | 1.00/0.02 | 0.95/0.15 |
| **rs2066845** | * | * | * | 0.47/0.001 | 0.96/0.07 |
| **rs2066847** | * | * | * | * | 0.93/0.19 |
| **rs2076756** | * | * | * | * | * |
