# Supplementary material for: The NOD2 Single Nucleotide Polymorphisms rs2066843 and rs2076756 Are Novel and Common Crohn's Disease Susceptibility Gene Variants
Source: PLoS One. 2010 Dec 30;5(12):e14466. doi: 10.1371/journal.pone.0014466 (PMC3012690; doi:10.1371/journal.pone.0014466)
Supplement: Table S7 — LD matrix for NOD2 SNPs in controls. Values are given as D'/r2. (0.03 MB DOC) [file pone.0014466.s007.doc]

**Supplemental Table S7.**

| ***NOD2* SNPs** | **rs2066843** | **rs2066844** | **rs2066845** | **rs2066847** | **rs2076756** |
| --- | --- | --- | --- | --- | --- |
| **rs2066843** | * | 0.90/0.11 | 0.95/0.05 | 0.86/0.05 | 0.94/0.82 |
| **rs2066844** | * | * | 1.00/0.001 | 0.09/<0.0001 | 0.09/0.84 |
| **rs2066845** | * | * | * | 0.99/0.001 | 0.89/0.05 |
| **rs2066847** | * | * | * | * | 0.96/0.07 |
| **rs2076756** | * | * | * | * | * |
