# Supplementary material for: The NOD2 Single Nucleotide Polymorphisms rs2066843 and rs2076756 Are Novel and Common Crohn's Disease Susceptibility Gene Variants
Source: PLoS One. 2010 Dec 30;5(12):e14466. doi: 10.1371/journal.pone.0014466 (PMC3012690; doi:10.1371/journal.pone.0014466)
Supplement: Table S8 — Haplotype-analysis for NOD2 SNPs in the UC patient cohort. Only omnibus p-values are presented, given that none of these haplotypes showed significant disease association. (0.02 MB DOC) [file pone.0014466.s008.doc]

**Supplemental Table S8.**

| ***NOD2* haplotypes** | **p-value** |
| --- | --- |
| rs2066843-rs2066844 | 0.660 |
| rs2066844-rs2066845 | 0.418 |
| rs2066845-rs2066847 | 0.425 |
| rs2066847-rs2076756 | 0.897 |
| rs2066843-rs2066844-rs2066845 | 0.323 |
| rs2066844-rs2066845-rs2066847 | 0.560 |
| rs2066845-rs2066847-rs2076756 | 0.598 |
| rs2066843-rs2066844-rs2066845-rs2066847 | 0.550 |
| rs2066844-rs2066845-rs2066847-rs2076756 | 0.669 |
| rs2066844-rs2066845-rs2066847-rs2076756-rs2076756 | 0.088 |
