# Supplementary material for: The NOD2 Single Nucleotide Polymorphisms rs2066843 and rs2076756 Are Novel and Common Crohn's Disease Susceptibility Gene Variants
Source: PLoS One. 2010 Dec 30;5(12):e14466. doi: 10.1371/journal.pone.0014466 (PMC3012690; doi:10.1371/journal.pone.0014466)
Supplement: Table S9 — Analysis for epistasis between SNPs rs2066843, rs2066843, rs2066844 (p.Arg702Trp), rs2066845 (p.Gly908Arg) and rs2066847 (p.Leu1007fsX1008) in the NOD2 gene and the SNP rs2241880 = p.Thr300Ala within the ATG16L1 gene regarding CD susceptibility. *All p values given are uncorrected for multiple comparisons. (0.02 MB DOC) [file pone.0014466.s009.doc]

**Supplemental Table S9.**

| ***NOD2* SNPs** | ***ATG16L1* rs2241880 (p.Thr300Ala)** |
| --- | --- |
| rs2066843 | 0.941 |
| rs2076756 | 0.990 |
| rs2066844 (p.Arg702Trp) | 0.734 |
| rs2066845 (p.Gly908Arg) | 0.425 |
| rs2066847 (p.Leu1007fsX1008) | 0.896 |
